# A Stability Training Method of Legged Robots Based on Training Platforms and Reinforcement Learning with Its Simulation and Experiment

**DOI:** 10.3390/mi13091436

**Published:** 2022-08-31

**Authors:** Weiguo Wu, Liyang Gao, Xiao Zhang

**Affiliations:** Humanoid & Gorilla Robot and Its Intelligent Motion Control Laboratory, School of Mechatronics Engineering, Harbin Institute of Technology, Harbin 150001, China

**Keywords:** legged robot, global self-stabilizer, stability training platform, Q-learning, composite disturbance, radial basis function network

## Abstract

This paper continues the proposed idea of stability training for legged robots with any number of legs and any size on a motion platform and introduces the concept of a learning-based controller, the global self-stabilizer, to obtain a self-stabilization capability in robots. The overall structure of the global self-stabilizer is divided into three modules: action selection, adjustment calculation and joint motion mapping, with corresponding learning algorithms proposed for each module. Taking the human-sized biped robot, GoRoBoT-II, as an example, simulations and experiments in three kinds of motions were performed to validate the feasibility of the proposed idea. A well-designed training platform was used to perform composite random amplitude-limited disturbances, such as the sagittal and lateral tilt perturbations (±25°) and impact perturbations (0.47 times the robot gravity). The results show that the proposed global self-stabilizer converges after training and can dynamically combine actions according to the system state. Compared with the controllers used to generate the training data, the trained global self-stabilizer increases the success rate of stability verification simulations and experiments by more than 20% and 15%, respectively.

## 1. Introduction

Compared with fix-based industrial robots, mobile robots have a wider application prospect because of their mobility and operational capacity. In particular, legged robots have a similar mechanism to animals and thus have a stronger adaptability to complex terrains than robots with other movements, such as wheeled and tracked robots. However, the practicality of legged robots is still lower than that of wheeled and tracked robots due to the difficulties in balance control and perturbation recovery.

The early studies mainly focused on the balance control of walking motions. Based on the Zero Moment Point (ZMP) force reflection control proposed by Vukobratovic [1], a variety of balance control methods such as body posture control [2], ZMP damping control [3] and landing point adjustment control [4] were proposed and deployed successively on ASIMO, Petman and other robots. Since then, researchers have started to consider the influence of perturbations and proposed corresponding control methods according to different types of perturbations. Successful results have been obtained for tilt ground [5,6,7], uneven ground [8], external force impact [9,10,11] and other perturbations.

The above balance controllers generate a planned response for a specific perturbation and then calculate the control outputs that enable the robot to track a determined trajectory by solving the dynamical model (or simplified model) of that robot. Thus, these controllers can be collectively defined as model-based balance controllers. Such controllers have achieved many successful results in structured environments such as laboratories, but their application is limited in unstructured, complex environments where the robot may be subject to multiple, mutually compounding, and unpredictable perturbations.

Consequently, more and more studies have paid attention to obtaining the self-stabilization capability of legged robots by using learning-based methods which are able to obtain the optimal mapping from the system state to the joint adjustment. The relevant literature on learning-based balance control methods is summarized in Table 1.

As shown in Table 1, some studies did not consider any perturbation, and the rest of them applied one kind of perturbation—generally a specific perturbation in a single direction. Moreover, the dimensions of the state/action space defined in existing studies are relatively low, which means that the learning process is carried out locally in the whole state space. In addition, the scope of state migration is relatively small when the applied perturbation is simple. Therefore, even though successful results can be obtained in the laboratory, state confusion is prone to occur in local state spaces determined by only partial state variables when the existing controllers face complex perturbations in reality, which leads to the failure in maintaining balance. In addition, the learning algorithms are applied without considering the curse of dimensionality when the number of state/action variables increases.

To address the above problems, the authors in [24] have proposed the idea of robot stability training—that is, to simulate composite perturbations by the random amplitude-limited motion of a six-degrees-of-freedom (DOF) training platform on which the robot is trained, and to obtain the self-stabilization capability by reinforcement learning with feature selection. A stability training simulation [25] of a bipedal robot was performed under randomly varying ground tilt perturbation, which preliminarily verified the feasibility of this idea. Relevant studies in medicine and biology also corroborate the practicability of stability training—for example, studies on movement disorder syndrome [26], stroke rehabilitation [27] and mice anatomy [28] have shown that training an organism with a moving platform can enhance or rebuild its balance.

In order to distinguish from the balance controllers learned under a single disturbance, the robot self-stabilizer trained on a 6-DOF motion platform called the global self-stabilizer, where “global” means that the training process has traversed all different kinds of environmental disturbances through the random amplitude-limited motion of the training platform. A robot self-stabilizer trained under such conditions can obtain robustness to any environmental perturbation, and after sufficient training, it can make the robot stable under any perturbation within its driving capability.

**Figure 1:**
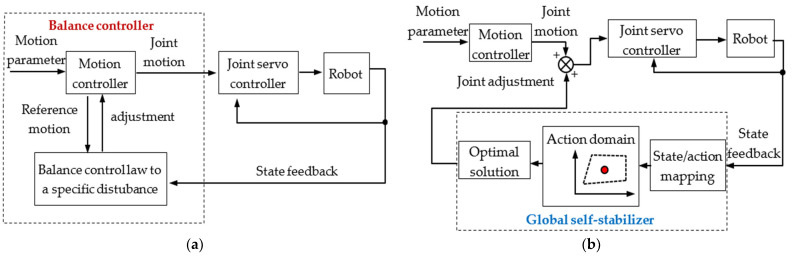
Comparison of a general balance controller and the global self-stabilizer for legged robots. (**a**) General balance controller; (**b**) global self-stabilizer.

Figure 1 compares the differences between the general balance controller of a legged robot and the global self-stabilizer in this study.

Figure 1a shows that a general robot balance controller uses a specific balance control law for different perturbations; the balance control of the robot is coupled with a specific motion, which means it is not universal.

As in Figure 1b, the global self-stabilizer is separated from the motion controller. The former finds the optimal joints’ increments according to the internal state/action map; the latter only needs to generate the reference motion according to the given motion parameters and is not affected by the global self-stabilizer. Thus, the two tasks (motion and balance) are independent. Only the target motion and the driving capability of the robot are considered in the motion controller. In other words, the self-stabilization capability obtained is not limited to specific motions, and the global self-stabilizer, such as a cerebellum, can be applied to any motion under any perturbation after being sufficiently trained.

In this paper, the stability training system of a legged robot with multiple legs is established and a general hierarchical structure of the global self-stabilizer is designed. The task of the proposed global self-stabilizer will be divided into three subtasks: action selection, adjustment calculation and joint motion mapping. Each subtask will be learned in different state spaces.

This paper is organized as follows: Section 2 describes the model of the training system and defines the state space of system variables and actions. Section 3 presents the three modules of the global self-stabilizer and their corresponding learning algorithms. Section 4 describes the simulated and experimental environments for stability training of a biped robot (GoRoBoT-II) and the balance controllers for generating training data. Section 5 and Section 6 presents the simulation and experiment training processes and results. This paper is concluded in Section 7.

## 2. A General Model for Stability Training of Legged Robots

**Figure 2:**
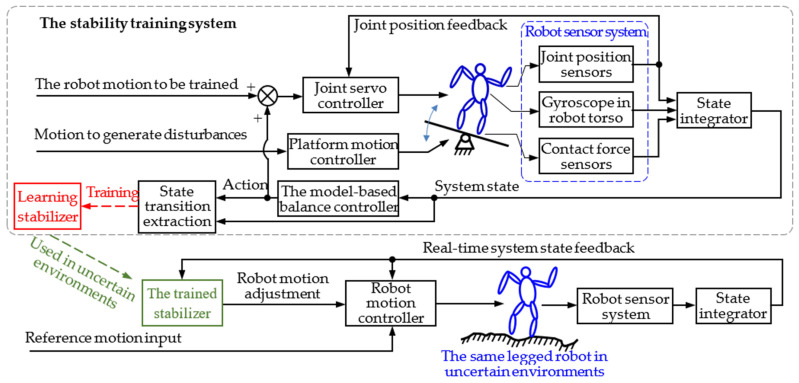
The basic idea of legged robot stability training and its application [25].

The basic idea of the legged robot stability training proposed by the authors is shown in Figure 2. During the training period, the robot stands on a training platform that performs a 6-DOF random amplitude-limited motion to simulate perturbations in the real world. The joint motion is generated by model-based balance controllers. The global self-stabilizer learns from the state transition data to obtain the optimal state/action mapping through reinforcement learning. After training, the converged global self-stabilizer can be used in uncertain environments to keep the robot stable.

### 2.1. Environmental Disturbance Simulation Method based on Motion Platform

**Figure 3:**
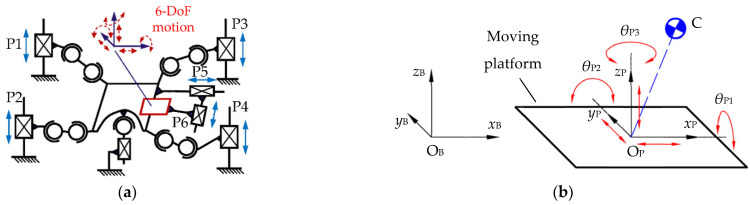
The mechanism of the training platform and its motion. (**a**) A 6-DOF serial–parallel mechanism of the training platform; (**b**) spatial motion of the training platform.

A dedicated 6-DOF serial-parallel mechanism motion platform [24,29] was designed in the authors’ laboratory for generating composite perturbations during stability training. Its mechanism sketch is shown in Figure 3a. The reference frames ΣO_B_-*x*_B_*y*_B_*z*_B_ and ΣO_P_-*x*_P_*y*_P_*z*_P_ are fixed to the ground and the platform, respectively. The motion of the moving platform can be represented by the displacements *x*_P_, *y*_P_, *z*_P_ and 3-2-1 Euler angles *θ*_P1_, *θ*_P2_ and *θ*_P3_ of the frame ΣO_P_ with respect to frame ΣO_B_ in Figure 3b. The pose vector can be expressed as ***X***_P_ = [*x*_P_, *y*_P_, *z*_P_, *θ*_P1_, *θ*_P2_, *θ*_P3_] ^T^. Point C represents the center of mass (CoM) of the trained robot.

Two forms of perturbations, ground tilt perturbations and inertial force/moment perturbations, can be generated by the above platform. If the training platform performs a random amplitude-limited motion, the generated tilt perturbation angle *β*, inertial force perturbation ***F***_P_ and inertial moment perturbation ***M***_P_ will also be randomly distributed within a certain range, thus enabling a comprehensive simulation of perturbations in the real world.

### 2.2. Model of the Training System

**Figure 4:**
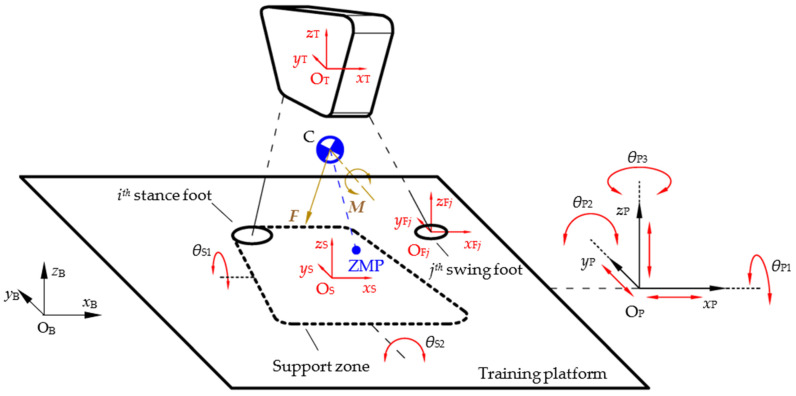
The general model for stability training system.

As shown in Figure 4, legged robots of any mechanical configurations and any size standing on the training platform can all be equated to a multi-branch chain rigid-body system with *n*_1_ (*n*_1_ ≥ 1) stance legs and *n*_2_ swing legs (*n*_2_ ≥ 0) if the motion in the air is not considered.

The reference frame ΣO_S_-*x*_S_*y*_S_*z*_S_ is established at the center of the theoretical support zone, and the motion of frame ΣO_S_ with respect to frame ΣO_P_ can represent the change in the contact state of the robot’s feet. In this study, situations in which the robot is completely in the air or the support foot slides on the training platform are not considered. Thus, only the 2-DOF flip motion of the theoretical support zone is analyzed, with the flip angles *θ_S1_* and *θ_S2_*, respectively. Each swing leg can be viewed as an open chain mechanism with its root located at the torso. The swing leg reference frame ΣO_F*j*_-*x*_F*j*_*y*_F*j*_*z*_F*j*_ is located at the center of the bottom surface of the *j*^*th*^ swing foot (*j* = 1, 2…*n*_2_). The motion of the swing leg can be represented by the pose vector ***X***_F*j*_—the pose of frame ΣO_F*j*_ with respect to the torso frame ΣO_T_-*x*_T_*y*_T_*z*_T_.

To establish the system variable set for the above model, the variables that can be measured or estimated in this system are summarized in Table 2.

For robots with any number of legs and any configuration, the system variable set can be constructed according to Table 2. In addition, the state variables corresponding to each action will be selected from the system variable set in the subsequent stability training.

### 2.3. Action Set of Legged Robot

The action set which stores the action variables and their adjustment equations is the discourse domain for the action selection. The action is considered as the active adjustment performed by the robot. So, after excluding the system variables that cannot be actively adjusted in the last two rows of Table 2, six types of actions are obtained: single-joint action, torso action, swing foot action, CoM action, inertial force/moment action and ZMP action (corresponding to the first six rows of Table 2, respectively).

In the stability training, the robot needs to accomplish three tasks simultaneously, i.e., tracking motion samples, resisting environmental (training platform) perturbations and avoiding joint limits. In the following, the six types of actions listed will be assigned to the three tasks mentioned above, and then the equation for action adjustment will be designed for each action. The parameters for each action are explained in Table 3.

(1) **Single-joint action.** When the robot’s joint reaches its position limit, velocity limit or acceleration limit, the motion of the robot will be affected, so joint limit avoidance is required.

The angular acceleration ${\ddot{\theta }}_{k}$ (*k* = 1, 2…*N*_J_) of the *N*_J_ joints of the robot are taken as the action variables in the single-joint action so that the motion curves obtained by integrating the acceleration are smoother than those obtained by directly adjusting the position and velocity. The adjustment is calculated according to Equation (1).

(1) $$\Delta {\ddot{\theta }}_{Xi}=L\left(\theta_{Xi},K_{11},\varepsilon_{11}\right)+L\left({\dot{\theta }}_{Xi},K_{12},\varepsilon_{12}\right)+L\left({\ddot{\theta }}_{Xi},K_{13},\varepsilon_{13}\right),\left(X=L,R{;}_{}i=1,2,\ldots ,6\right)$$

The compensation equation for the joint angular limit is calculated according to Equation (2). The compensation equations for joint velocity and acceleration are similar and will not be listed specifically.

(2) $$L\left(\theta_{Xi},K_{11},\varepsilon_{11}\right)=\left\{\begin{array}{ll} -K_{11}\left(\theta_{Xi}-\theta_{Xi}^{\max}+\varepsilon_{1}\right), & \theta_{Xi}>\theta_{Xi}^{\max}-\varepsilon_{1}\\ 0, & O.W.\\ K_{11}\left(\theta_{Xi}^{\min}+\varepsilon_{1}-\theta_{Xi}\right), & \theta_{Xi}<\theta_{Xi}^{\min}+\varepsilon_{1} \end{array}\right.$$

(2) **Torso action**. This kind of action is used to bring the robot stance leg back to the preset motion sample after other adjustments. The action variable is chosen as ${\ddot{X}}_{T}$, and its adjustment is calculated using the PD control law shown in the following equation.

(3) $$\Delta {\ddot{X}}_{T}={ [\begin{array}{cccccc} \Delta {\ddot{x}}_{T} & \Delta {\ddot{y}}_{T} & \Delta {\ddot{z}}_{T} & \Delta {\ddot{\theta }}_{T1} & \Delta {\ddot{\theta }}_{T2} & \Delta {\ddot{\theta }}_{T3} \end{array}]}^{T}=K_{21}\left(X_{T}^{d}-X_{T}\right)+K_{22}\left({\dot{X}}_{T}^{d}-{\dot{X}}_{T}\right)+{\ddot{X}}_{T}^{d}$$

(3) **Swing foot action**. Similar to the torso action, for the *n*_2_ swimming feet in the general model. The action variables are chosen as ${\ddot{X}}_{Fj}$ (*j* = 1, 2…*n*_2_) and the adjustment is calculated using the PD control law shown in the following equation.

(4) $$\Delta {\ddot{X}}_{Fj}={ [\begin{array}{cccccc} \Delta {\ddot{x}}_{Fj} & \Delta {\ddot{y}}_{Fj} & \Delta {\ddot{z}}_{Fj} & \Delta {\ddot{\theta }}_{Fj1} & \Delta {\ddot{\theta }}_{Fj2} & \Delta {\ddot{\theta }}_{Fj3} \end{array}]}^{T}=K_{31}\left(X_{Fj}^{d}-X_{Fj}\right)+K_{32}\left({\dot{X}}_{Fj}^{d}-{\dot{X}}_{Fj}\right)+{\ddot{X}}_{Fj}^{d}$$

(4) **CoM action.** This type of action will directly adjust the robot CoM to keep balance on the moving platform. The action variable is chosen as the linear acceleration of the CoM. To keep the CoM above the stance legs, the adjustment is calculated according to the estimated position of the moving platform.

(5) $${ [\begin{array}{ccc} \Delta {\ddot{x}}_{C} & \Delta {\ddot{y}}_{C} & \Delta {\ddot{z}}_{C} \end{array}]}^{T}=K_{41}\left(P_{S}+{ [\begin{array}{ccc} 0 & 0 & l_{C} \end{array}]}^{T}-P_{C}\right)+K_{42}\left({\dot{P}}_{S}-w_{P}\times { [\begin{array}{ccc} 0 & 0 & l_{C} \end{array}]}^{T}-{\dot{P}}_{C}\right)-{\ddot{P}}_{C}$$

(5) **Inertial force/moment action.** The inertial forces and moments influenced by the motion of the limbs are taken as a class of actions to cope with the perturbations. The action variables are chosen as the inertial force ***F*** and the inertial moment ***M*** at the CoM. The kinetic energy attenuation method proposed by the authors of [11] is used here to keep the robot balanced. The adjustment is calculated as follows:

(6) $$\begin{array}{l} { [\begin{array}{ccc} \Delta F_{X} & \Delta F_{Y} & \Delta F_{Z} \end{array}]}^{T}=F-F_{\mathrm{last}}=K_{51}m_{C}v_{C}-F_{\mathrm{last}}\\ { [\begin{array}{ccc} \Delta M_{X} & \Delta M_{Y} & \Delta M_{Z} \end{array}]}^{T}=M-M_{\mathrm{last}}=K_{52}L_{C}-M_{\mathrm{last}} \end{array}$$

(6) **ZMP action.** As a common control strategy in robot balance control, changing the ZMP position within the support zone through limb motion can be used as a class of action in response to perturbations. Therefore, the action variables are chosen as *x*_ZMP_ and *y*_ZMP_. Using the pose balance control method based on the CP point proposed by the authors of [8], the ZMP adjustment is calculated with the following equation:

(7) $${ [\begin{array}{cc} \Delta x_{\mathrm{ZMP}} & \Delta y_{\mathrm{ZMP}} \end{array}]}^{T}=\left({1+K}_{6}\right)P_{\mathrm{CP}}-K_{6}P_{0}-P_{\mathrm{ZMP}}$$

The action set of legged robots can be written as:

(8) $$Q=\left\{\Delta {\ddot{\theta }}_{1}\;\Delta {\ddot{\theta }}_{2}\cdots {\ddot{\theta }}_{N_{J}}\;\Delta {\ddot{X}}_{T}\;\Delta {\ddot{X}}_{F1}\;\Delta {\ddot{X}}_{F2}\cdots \Delta {\ddot{X}}_{Fn_{2}}\;\Delta {\ddot{P}}_{C}\;\Delta F\;\Delta M\;\Delta x_{\mathrm{ZMP}}\;\Delta y_{\mathrm{ZMP}}\right\}$$

Although only one equation is given for the adjustment of each action in ***Q***, different adjustments can be obtained by adjusting the 12 free parameters (*K*_11_, *K*_12_, *K*_13_, *K*_21_, etc.). The determination methods and specific values of these parameters will be illustrated in Section 4 with simulation examples.

## 3. The Global Self-Stabilizer

### 3.1. Preprocessing and Structure of the Global Self-Stabilizer

Dimensionality reduction and discretization are required to enable the learning process to exponentially converge because the system space designed in Section 2.2 is a high-dimensional continuous space. The system variable set listed in Table 2 is denoted as ***X*** = {*x_i_* |*I* = 1, 2…*N*}, and the action set in 2.3 is denoted as ***Q*** = {*q_j_* |*j* = 1, 2…*m*}. The global self-stabilizer in this study will establish the mapping from ***X*** to ***Q***.

The RAFS feature selection method proposed by the authors in [30] will be used to reduce the dimensionality of the system space to obtain the state set $S_{j}=\left\{\left.s_{jk}\right|k=1,2,\cdots ,N_{Sj};s_{jk}\in X\right\}$—corresponding to each action *q_j_* and followed by the autonomic abstraction calculation of the state space based on the Gaussian basis functions proposed by the authors in [25]. The continuous state space corresponding to ***S****_j_* is then discretized into different Gaussian basis functions according to the maximum affiliation principle. The full set of Gaussian basis functions corresponding to ***S****_j_* can be expressed as ***Ψ****_j_* = {***ψ****_jk_* = <***μ****_jk_*, **Σ***_jk_*>*|k* = 1, 2, …, *N*_B*j*_}, where ***μ****_jk_* and **Σ***_jk_* are the center vector and covariance matrix of the basis function ***ψ****_jk_*, respectively.

With ***x*** = [*x*_1_, *x*_2_…*x_N_*] ^T^ denoting the vector in the system space and ***s****_j_* = [*s_j_*_1_, *s_j_*_2_…*s_jN_*_S*j*_] ^T^ denoting the vector in the state space of action *q_j_*, the mapping of ***X*** to ***S****_j_* after feature selection can be expressed as:

(9) $$s_{j}=Wx$$

where ***W****_j_* is the *N*_S*j*_ × *N* selection matrix obtained from the feature selection calculation.

The affiliation of the reduced-dimensional state vector ***s****_j_* to the basis function ***ψ****_jk_* can be expressed as:

(10) $$f\left(s_{j},\psi_{jk}\right)=e^{-0.5{\left(\mu_{jk}-s_{j}\right)}^{T}\Sigma_{jk}^{-1}\left(\mu_{jk}-s_{j}\right)}$$

The *N*_S*j*_-dimensional continuous state space corresponding to ***S****_j_* can thus be transformed into a discrete space with *N*_B*j*_ values. To facilitate the learning calculation of the global self-stabilizer in Section 2, a normalized affiliation function is also defined.

(11) $$\hat{f}\left(s_{j},\psi_{jk}\right)=f\left(s_{j},\psi_{jk}\right)/\sum_{i=1}^{N_{Bj}}f\left(s_{j},\psi_{ji}\right)$$

The legged robot’s actions need to be executed by the joint motion, so the global self-stabilizer also needs to establish the mapping from ***Q*** to the joint angular acceleration increment vector $\Delta \ddot{\theta }={ [\theta_{1},\theta_{1},\cdots ,\theta_{N_{J}}]}^{T}$. Because the action variables in ***Q*** are all acceleration or force/moment, we can linearize the kinematic or dynamical equations of the system:

(12) $$q_{j}=b_{j}\cdot \Delta \ddot{\theta }\begin{array}{cc} & \left(j=1,2,\cdots ,m\right) \end{array}$$

where ***b****_j_* is the *N*_J_-dimensional joint motion mapping vector, which represents the projection of the action adjustment *q_j_* in the robot joint space. Combining the joint motion mapping vectors into the mapping matrix ***B*** = [***b***_1_, ***b***_2_, …, ***b****_n_*] ^T^, Equation (12) can then be written as:

(13) $$q=B\Delta \ddot{\theta }$$

In general, the number of actions *m* is greater than the robot DOF *N*_J_, so the matrix ***B*** is singular. Therefore, the action selection matrix ***A****_N_*_J×*m*_ is constructed, which has only one element of 1 in each row and the remaining elements of 0. Adding the action selection matrix ***A*** to Equation (13) gives:

(14) $$\Delta \ddot{\theta }={\left(AB\right)}^{-1}Aq$$

**Figure 5:**
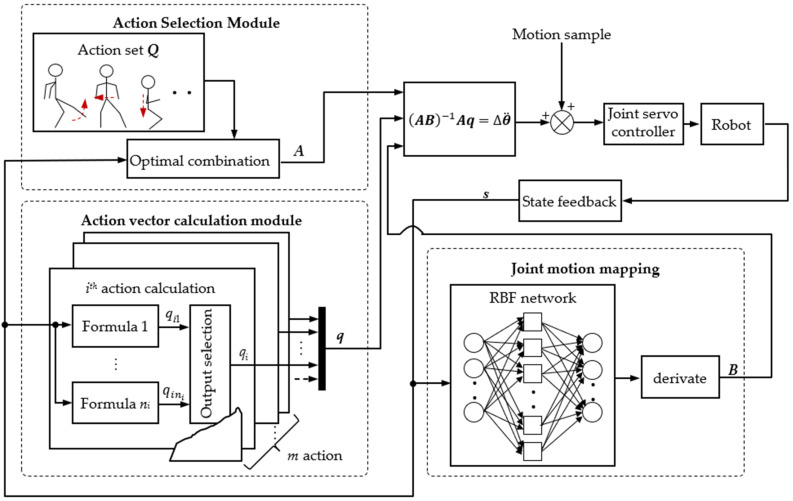
Structure of global self-stabilizer.

According to Equation (14), the global self-stabilizer is divided into three modules in this study: action selection module, adjustment calculation module and joint motion mapping module, which are used to generate ***A***, ***q*** and ***B***, respectively. The specific structure of the global self-stabilizer is shown in Figure 5.

### 3.2. Action Selection Module

The action selection module selects *N*_J_ actions in the action set and generates the action selection matrix ***A***. Two main considerations are made when selecting the combination of actions: the value of the actions for the robot stability at the current state, and the influence of the actions on each other when combined.

The action value function is defined as ***V***_A_(***x***). The mutual influence between actions is shown by the singularity of ***AB*** in Equation (14). For the action variables *q_i_* and *q_j_*, the mutual influence $c_{ij}$ is quantified by the relative projection of the joint mapping vectors ***b****_i_* and ***b****_j_* (defined in Section 3.4):

*c_ij_* = |***b****_i_*^T^***b****_j_*|/(||***b****_i_*||×||***b****_j_*||), (15)

For any action selection of matrix ***A***, we can define the action selection evaluation function as follows:

(16) $$E_{A}(A,x)=V_{A}^{}\left(x\right)\cdot \left(A^{T}1\right)-\omega_{C}{\left(A^{T}1\right)}^{T}C\left(A^{T}1\right)$$

where 1 is an all-one vector, and *ω*_C_ is the weight of the action value and mutual influence. Action selection can be achieved by solving the optimization model shown in Equation (17).

(17) $$\begin{array}{l} A=\arg\max E_{A}(A,x)\\ \begin{array}{c} s.t.\mathrm{rank}(A)=N_{J} \end{array} \end{array}$$

### 3.3. Adjustment Calculation Module

There may be different formulas (or different parameters) for calculating the adjustment of the same action because the training data of the global self-stabilizer may have multiple sources (model-based controllers, motion capture data, etc.). Therefore, the task of the adjustment calculation module is to select the most valuable adjustment calculation formula for each action, then calculate and output action adjustment ***q***.

Assuming that the *j*^*th*^ action has *n_j_* (*j* = 1, 2, …, *m*) different adjustment formulas, the value functions of these formulas *V_jk_*(***x***) (j = 1, 2, …, m; k = 1, 2, …, *n*_*j*_) are obtained by learning, and the formula with the largest *V_jk_*(***x***) is selected to calculate *q_j_*.

The action value function *V*_A*j*_(***x***) can be determined by *V_jk_*(***x***):

(18) $$V_{Aj}\left(x\right)=\underset{k=1,2,\ldots ,n_{j}}{\max}V_{\;jk}\left(x\right)$$

Both the action selection module and the adjustment calculation module need to determine the value function *V_jk_*(***x***) through learning. The learning of *V_jk_*(***x***) will be introduced below. The state transition of the training data at each moment can be extracted as a quintuple <***x***, ***I***, $\Delta \ddot{\theta }$, *r*, ***x***′>, where ***I*** is the activation flag matrix of the adjustment calculation formula, and the element *I_jk_* takes 1 when the adjustment *q_j_* is calculated by the *k*^*th*^ formula. ***x***′ is the system variable vector at the next moment. *r* is the immediate reward, considering the stability of the robot and the difference between the actual motion and the reference motion of the robot. The reward function *r* is defined according to Equation (19).

(19) $$r=\left\{\begin{array}{l} \begin{array}{c} \frac{1}{n}\sum_{i=1}^{N_{J}}\left(1-\frac{\left|\theta_{i}^{d}-\theta_{i}\right|}{\theta_{i\max}-\theta_{i\min}}\right),\;\mathrm{stable} \end{array}\\ \begin{array}{c} -100,\;\mathrm{unstable} \end{array} \end{array}\right.$$

where *θ_i_*^d^ is the joint angle of the *i*^th^ joint in the motion sample; *θ_i_*_max_ and *θ_i_*_min_ are the positive and negative limit positions of the *i*^th^ joint, respectively.

In this paper, ZMP is not the only criterion for determining stability. When ZMP is within the support zone, the robot is considered to be stable; when ZMP exceeds the support zone, the robot will start to flip along the boundary of the support zone. The robot is still considered to have the possibility of recovery when the flip angle is less than 45°; only after the flip angle exceeds 45° is the robot considered to be in an irrecoverable unstable state.

For each training data, the value function *Q*(***ψ****_ijk_*) is updated by Q-learning.

(20) $$Q\left(\psi_{ijk}\right)\leftarrow Q\left(\psi_{ijk}\right)+I_{jk}\alpha [r_{jk}f\left(x,\psi_{ijk}\right)+\gamma \sum_{h=1}^{N_{Bjk}}f\left(x^{\prime },\psi_{hjk}\right)Q\left(\psi_{hjk}\right)-Q\left(\psi_{ijk}\right)]$$

where *r_jk_* is the reward function after assigning the immediate reward *r* to the *k*^*th*^ adjustment calculation formula for the *j*^*th*^ action, calculated according to Equation (21).

(21) $$r_{jk}=\frac{rI_{jk}\left|b_{j}\cdot \Delta \ddot{\theta }\right|/\left\|b_{j}\right\|}{\sum_{j=1}^{m}\sum_{k=1}^{n_{j}}\left(I_{jk}\left|b_{j}\cdot \Delta \ddot{\theta }\right|/\left\|b_{j}\right\|\right)}$$

### 3.4. Joint Motion Mapping Module

The task of this module is to give the joint mapping matrix ***B*** based on the feedback of the system variable ***x***. The radial basis function network (RBF network) will be used to train the mapping relation $(x,\Delta \ddot{\theta })\to q$ as an approximation to the system motion equations because it is difficult to obtain training data in the form of <***x***, ***B***> directly. However, it is always possible to extract training data in the form of <***x***, ***q***, $\Delta \ddot{\theta }$> from the robot state transition. Then, the local linearized mapping matrix ***B*** is obtained by differentiating this RBF network.

This network is split into sub-networks with one single behavioral variable *q_i_* to reduce the complexity. In Equation (12), ignoring the effect of $\Delta \ddot{\theta }$ on ***b****_i_*, the mapping vector ***b****_i_* is considered as a function of ***x*** only. After performing the local linearization, *q_i_* can be calculated by the following equation.

(22) $$q_{i}=q_{i0}+b_{i0}^{T}\left(\Delta \ddot{\theta }-\Delta {\ddot{\theta }}_{0}\right)$$

where *q_i_*_0_, ***b****_i_*_0_ $\Delta {\ddot{\theta }}_{0}$ are the mean values of *q_i_*, ***b****_i_* and $\Delta {\ddot{\theta }}_{0}$ in the neighborhood of the local linearization, respectively.

**Figure 6:**
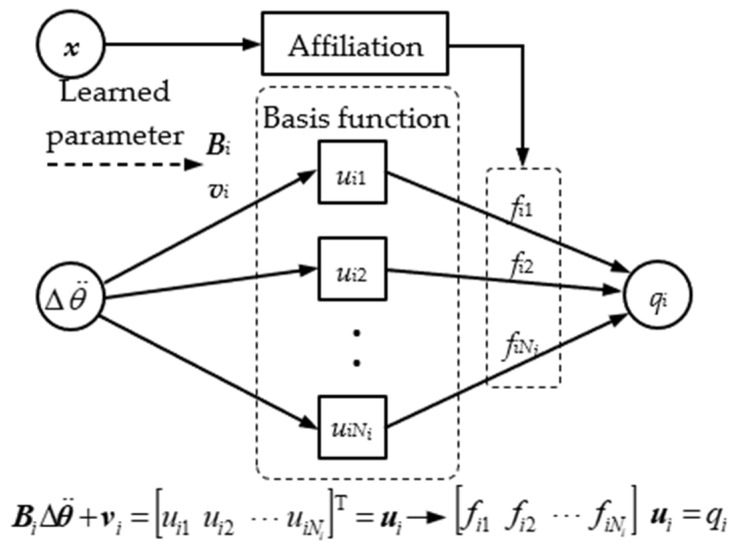
Structure of the RBF network.

The RBF network structure is shown in Figure 6, where ***B****_i_* and ***v****_i_* are the weight matrix and bias vector connecting the input layer to the hidden layer, respectively; *u_i_*_j_ (*I* = 1, 2, …, m; j = 1, 2, …, *N_i_*) is the linear activation function; the connection weights of the hidden layer to the output layer are the affiliation function *f_ij_* (defined in Equation (11)). The output equation of the network is shown in Equation (23), where ***f****_i_* = [*f_i_*_1_, *f_i_*_2_, …, *f_i_*_N*i*_]^T^.

(23) $$q_{i}=f_{i}^{T}\left(B_{i}\Delta \ddot{\theta }+v_{i}\right)$$

The basis functions of the above RBF network are evaluated only in the space tensorized by x to reduce the number of basis functions. This modified RBF network is equivalent to linear (first order) interpolation in the multi-dimensional space, which can improve the fitting accuracy.

Differentiating Equation (23), the equation for the mapping vector ***b****_i_* extracted from the RBF network is:

(24) $$b_{i}=\frac{\partial q_{i}}{\partial \Delta \ddot{\theta }}=B_{i}^{T}f_{i}$$

The training of the designed RBF network is divided into two steps: (1) determination of the center and boundary of the basis function; (2) local training inside the basis function.

The center and boundary of the basis function are determined by the state space autonomic abstraction calculation based on the Gaussian base function [25] (feature selection is also required). For each RBF sub-network, the basis function set can be expressed as ***Ψ***_B*i*_ = {***ψ***_B*ij*_*|j* = 1, 2, …, *N_i_*} after the autonomic abstraction calculation.

For the *j*^*th*^ basis function of action *q*_i_, the following error function can be defined:

(25) $$e_{ij}=\frac{1}{2}\sum_{k=1}^{N_{qi}}f\left(s_{i}^{\left(k\right)},\psi_{Bij}\right){\left(q_{i}^{\left(k\right)}-b_{Rij}\Delta {\ddot{\theta }}^{\left(k\right)}-v_{ij}\right)}^{2}$$

The superscript (k) represents the *k*^*th*^ training data, ***b***_R*ij*_ is the *j*^*th*^ row of the weight matrix ***B****_i_*, and *v_ij_* is the *j*^*th*^ element of the bias vector ***v****_i_*. For simplicity, *f*(***s****_i_*^(*k*)^, ***W***_B*i*_, ***ψ***_B*ij*_) is abbreviated as *f_ijk_*. To minimize *e_ij_*, the following equations need to be solved:

(26) $$\frac{\partial e_{ij}}{\overset{}{\partial } [\begin{array}{cc} b_{Rij} & v_{ij} \end{array}]}=\sum_{k=1}^{N_{qi}}\left\{f_{ijk}\left(b_{Rij}\Delta {\ddot{\theta }}^{\left(k\right)}+v_{ij}-q_{i}^{\left(k\right)}\right){ [\begin{array}{c} \Delta {\ddot{\theta }}^{\left(k\right)}\\ 1 \end{array}]}^{T}\right\}=0$$

Solving Equation (26), the solution shown in Equation (27) can be obtained.

(27) $${ [\begin{array}{cc} b_{Rij} & v_{ij} \end{array}]}^{T}={\left(\hat{U}U_{}^{T}\right)}^{-1}\left(\hat{U}q_{Li}\right)$$

Where the definition of ***U,*** $\hat{U}$ and ***q***_L*i*_ are shown in:

(28) $$U= [\begin{array}{cccc} \Delta {\ddot{\theta }}^{\left(1\right)} & \Delta {\ddot{\theta }}^{\left(2\right)} & \cdots & \Delta {\ddot{\theta }}^{\left(N_{qi}\right)}\\ 1 & 1 & \cdots & 1 \end{array}]$$

(29) $$\hat{U}= [\begin{array}{cccc} f_{ij1}\Delta {\ddot{\theta }}^{\left(1\right)} & f_{ij2}\Delta {\ddot{\theta }}^{\left(2\right)} & \cdots & f_{ijN_{qi}}\Delta {\ddot{\theta }}^{\left(N_{qi}\right)}\\ f_{ij1} & f_{ij2} & \cdots & f_{ijN_{qi}} \end{array}]$$

(30) $$q_{Li}={ [\begin{array}{cccc} q_{i}^{\left(1\right)} & q_{i}^{\left(2\right)} & \cdots & q_{i}^{\left(N_{qi}\right)} \end{array}]}^{T}$$

## 4. Stability Training System of Biped Robots

Taking the biped robot GoRoBoT-II as an example, the simulated and experimental stability training environment are established to validate the effectiveness of the proposed idea and the balance controllers for generating the training data are designed.

### 4.1. Simulation Environment

**Figure 7:**
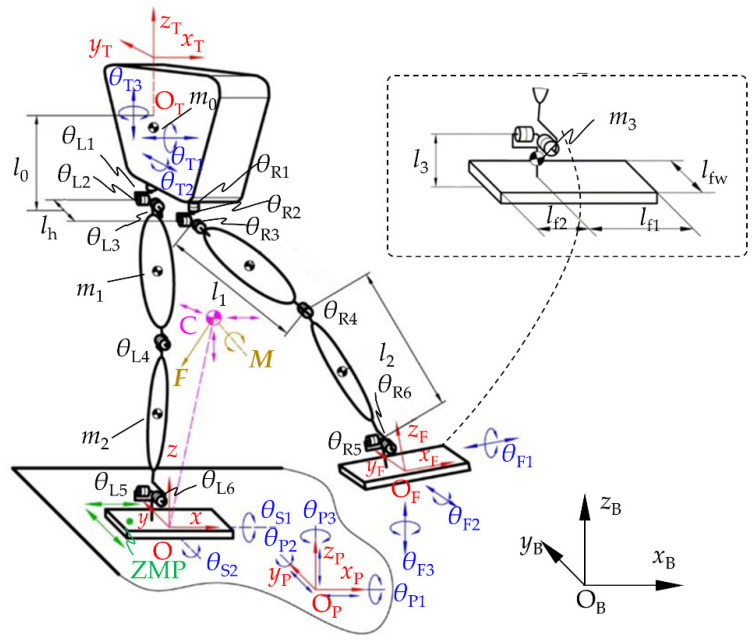
Multi-rigid-body model of the biped robot.

The biped robot used in this study is the bipedal part of the GoRoBoT-II robot designed by the author’s laboratory. Its main mechanism parameters are shown in Table 4. The seven-bar multi-rigid-body model of the biped robot is shown in Figure 7. The reference frames and variables are defined according to the model in Section 2.2. In addition, the joint angles of the left and right legs are denoted as *θ*_L*i*_ and *θ*_R*i*_ (i = 1, 2, …, 6), respectively.

### 4.2. Experiment Environment

**Figure 8:**
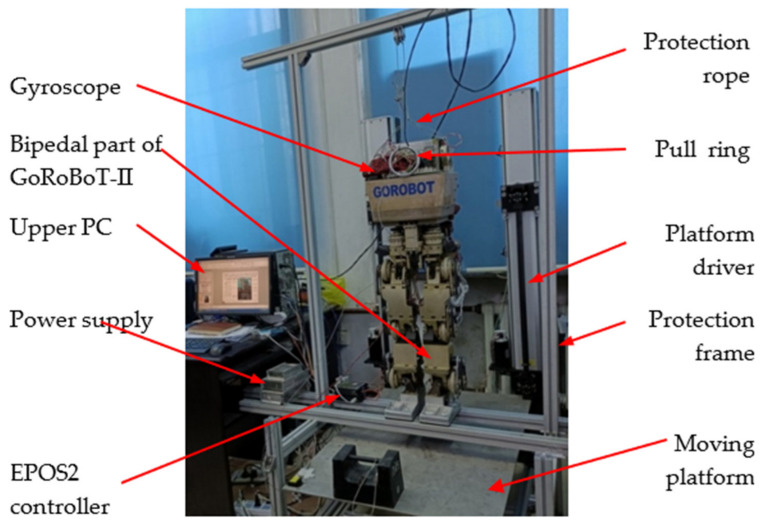
Biped robot stability training experiment system.

The experimental system for stability training is shown in Figure 8, which includes the upper computer, motion platform, biped robot and protection device. The upper computer is a PC with a Windows operating system. The protection device is composed of a wire rope, a fixed pulley and a pull ring. When the robot is stable, the wire rope stays slack and does not affect the robot; when the robot is unstable, the experimenter pulls the protection rope tightly to prevent the robot from falling down.

**Figure 9:**
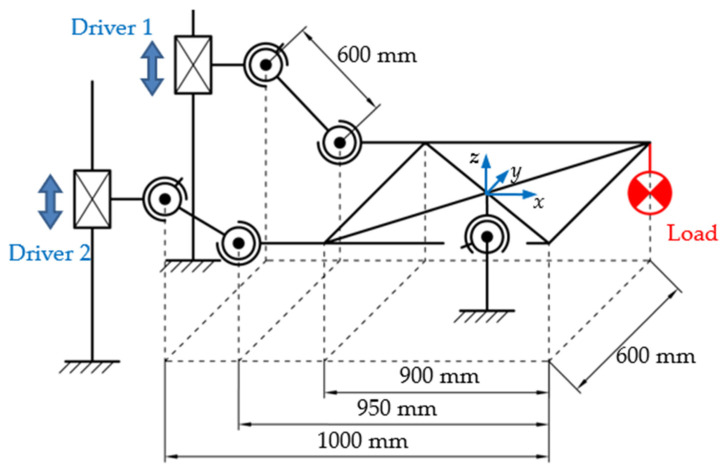
Mechanism diagram of the 2-DOF motion platform.

The training platform in the above system is a 2-DOF motion platform. The mechanism diagram and its main parameters are given in Figure 9. The motion platform can oscillate around the *x*-axis and *y*-axis, denoted by *θ*_P1_ and *θ*_P2_, respectively. The limits of oscillation amplitude, speed and acceleration are ±20°, ±40°/s and ±60°/s^2^, respectively, which meet the requirements for stability training.

Each joint of the robot is driven by a Maxon RE35 DC servo motor. The transmission system consists of a synchronous belt drive (first stage) and a harmonic gear drive (second stage).

The motion control commands of the robot are generated by the upper computer, and the DC servo motors of each joint are position servos controlled by IPM100 controllers. In addition to the photoelectric encoders on the DC servo motors, the robot is equipped with a gyroscope (mounted on the torso) and force sensors (mounted under the soles of the feet) to measure the acceleration and velocity of the torso as well as the contact forces, respectively.

### 4.3. Balance Controllers for Stability Training Data Generation

The model-based balance controllers used for training data generation can be obtained by combining actions in action set ***Q***. The stance leg can follow the motion sample input when the behavior variable ${\ddot{X}}_{T}$ is adjusted according to Equation (3); similarly, when ${\ddot{X}}_{F}$ is adjusted according to Equation (4), the swing leg can follow the motion sample input. Thus, if the robot’s action vector is chosen to be ${ [\begin{array}{cc} {\ddot{X}}_{T}^{T} & {\ddot{X}}_{F}^{T} \end{array}]}^{T}$, the robot’s motion will be completely limited to the motion sample input.

By replacing some elements of the above action vector with the variables of three types of actions—CoM action, inertial force/moment action and ZMP action—the balance adjustments can be achieved based on the input sample motions. A variety of legged robot balance controllers with different action combinations can be obtained. The three types of controllers are described in detail below.

(1) **CoM adjustment balance controller.** This controller maintains the robot’s balance by keeping **the** robot’s CoM above its support zone. The action variable that must be selected is ${\ddot{P}}_{C}$, and the action variable to be replaced can be ${\ddot{x}}_{T}$, ${\ddot{y}}_{T}$ or ${\ddot{z}}_{T}$ in ${\ddot{X}}_{T}$, and ${\ddot{x}}_{F}$, ${\ddot{y}}_{F}$ or ${\ddot{z}}_{F}$ in ${\ddot{X}}_{F}$. The former corresponds to adjusting the robot’s CoM by translational motion of the torso, and the latter by the swing foot.

(2) **Energy attenuation balance controller.** This controller dissipates the system energy by making the inertial force and moment do negative work, thus achieving stabilization. The action variables to be selected are ***F*** and ***M***. The action variables that can be removed are the torso acceleration ${\ddot{X}}_{T}$ or the swing leg acceleration ${\ddot{X}}_{F}$, which correspond to the two ways of changing the inertial force and moment by the stance leg adjustment or the swing leg adjustment, respectively.

(3) **ZMP adjustment balance controller.** This controller keeps the robot’s CP point in the center of the support zone by adjusting the ZMP. Therefore, the action variables that must be selected are *x*_ZMP_ and *y*_ZMP_, and the substituted action variables can be ${\ddot{x}}_{T}$ or ${\ddot{y}}_{T}$ in ${\ddot{X}}_{T}$, and ${\ddot{x}}_{F}$ or ${\ddot{y}}_{F}$ in ${\ddot{X}}_{F}$, which is equivalent to the adjustment of the ZMP position by torso swing or swing leg kick.

Table 5 summarizes the six balance controllers. During the stability training, the action selection matrix ***A*** is determined by the corresponding controllers used in Table 5; the joint mapping matrix ***B*** is calculated according to the kinematics and dynamics of the robot; the adjustment vector Δ***q*** within each control cycle is calculated from the corresponding adjustment calculation formula (Equations (1)–(7)) according to the current state x; and the control output $\Delta \ddot{\theta }$ is solved by Equation (14). Furthermore, the state transition information <***x***, ***I***, $\Delta \ddot{\theta }$, *r*, ***x***′> generated by the above balance controller will be recorded to form the training data, and this data will be used for learning the three modules of the global self-stabilizer.

When the position, velocity or acceleration of a joint enters its limit neighborhood (determined by *ε*_J1_, *ε*_J2_ and *ε*_J3_), the joint limit will be avoided by the single-joint action, which is achieved by selecting one of the single-joint actions that has the largest influence coefficient (defined by Equation (15)).

## 5. Simulation Results

Here, the stability training data of the single-leg stance, double-leg stance and stepping will be generated within the simulation environment established in 4.1 using the model-based balance controllers in 4.3 to train the global self-stabilizer, after which the stability verification simulation of the trained global self-stabilizer will be performed under the same conditions.

### 5.1. Stability Training in Simulation

In the stability training simulation, the motion platform applies two kinds of perturbations. The first one is time-varying ground tilt perturbation by the amplitude-limited random motion of the swing angle *θ*_P1_ and *θ*_P2_ (see Figure 7); the other is the impact perturbation by the sudden change of angular velocity based on the first one.

Three different sets of the control parameters in the adjustment amount (Equations (1)–(7)) are designed, corresponding to different response speeds. The specific values are given in Table 6, which were obtained from the simulation conducted before training. The superscript is used to indicate the level action that the variable takes, such as *x*_ZMP_^(1).^

Three reference motions were used for the stability training simulation, i.e., single-leg stance, double-leg stance and stepping. The stepping motion has random landing points, and the motion samples were obtained by the planning method proposed in [31]. One hundred simulations were performed for each level of each balance controller under each perturbation condition in Adams, and 4000 system variable transition data were extracted from each simulation. The duration of each simulation was 20 s, and the control period was 5 ms. For the controllers of TC*_i_*, TE*_i_* and TB*_i_* (*I* = 1, 2, 3), a total of 1.2 × 10^6^ transition data of system variables without impact and 8 × 10^5^ with impact were obtained, respectively; for the controllers of FC*_i_*, FE*_i_* and FB*_i_* (*I* = 1, 2, 3), a total of 8 × 10^5^ transition data of system variables without impact and 4 × 10^5^ with impact were obtained, respectively. The maximum simulation success rates among all model-based controllers are shown in Table 8.

As a preparation for Q-learning and RBF network learning, feature selection and autonomic abstraction calculations were performed first, and the results are shown in Table 7. The value functions of the actions with different parameters share the same feature selection results, but the state space autonomic abstraction calculation is performed with different basis function distributions so that different parameters obtain different numbers of basis functions.

A total of 198 system variables were selected for the 40 functions in the above table. There were an average of five state variables per function from 113 system variables, which shows that the RAFS feature selection method effectively reduces the state space dimensionality of learning.

**Figure 10:**
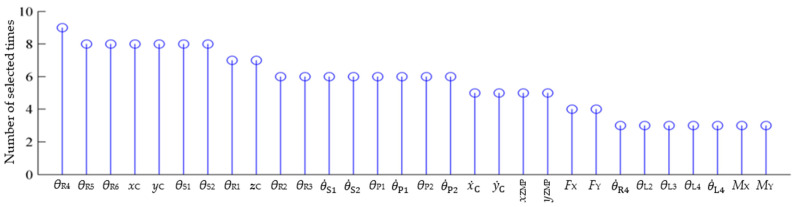
The 30 most-selected system variables.

The 30 most-selected system variables are shown in Figure 10. The most-selected variables are joint angles of stance leg, followed by the position of CoM and the flip angle. Overall, the system variables related to robot CoM, platform swing angle, resultant force/moment and ZMP are all present in the top 30 most-selected variables. All of these are important variables or equilibrium criteria in biped robot balance control, which indicates that the RAFS feature selection method successfully selected system variables of significance.

The 40 functions in Table 7 were learned separately after the feature selection and state space autonomic abstraction calculation described above. The Q-learning of the action values was trained in a batch, with the amount of training data for each batch being 10,000, and the incremental threshold of the value function for iterative convergence set to 10^−5^; the RBF network for joint motion mapping was trained according to Equation (27). The optimal solution was converged after performing one iteration on all training data.

### 5.2. Stability Verification Simulation of the Trained Global Self-Stabilizer

To verify the effectiveness of the trained global self-stabilizer, five hundred stability verification simulations were performed on the motion platform for each of the three robot motions, under the same simulation conditions and parameters as the training data generation. The success rates of the above verification simulations are presented in Table 8 and are compared with the highest success rate of the model-based balance controllers.

From the above table, it can be seen that the trained global self-stabilizer obtains stronger stability than the model-based balance controllers, with increases ranging from around 10% to 33%. The global self-stabilizer nearly doubles the success rate when the impact perturbations are applied.

The verification simulation results of the single-leg stance and the stepping will be analyzed next, because the double-leg stance is less challenging than others.

**Figure 11:**
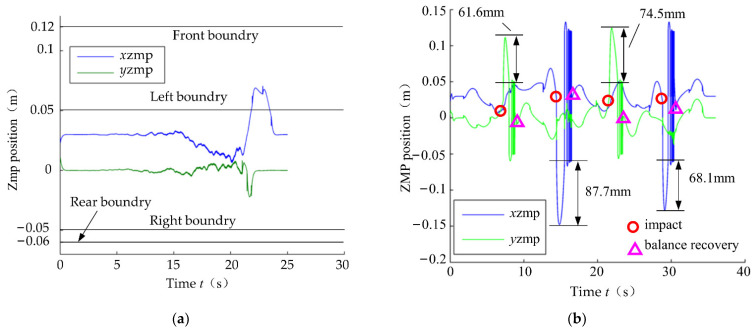
ZMP curves in two single-leg stance simulations. (**a**) Without impact; (**b**) with impact.

The ZMP curves in two single-leg stance simulations are given in Figure 11, respectively. Figure 11a depicts that the trained global self-stabilizer regulated the ZMP to the center of the support zone when no impact perturbation is applied. Figure 11b shows that the ZMP exceeded the support zone boundary with the farthest distance of 87.7 mm after the impact, and the global self-stabilizer reduced the ZMP’s oscillation amplitude and finally recovered the flat-foot contact of the robot.

**Figure 12:**
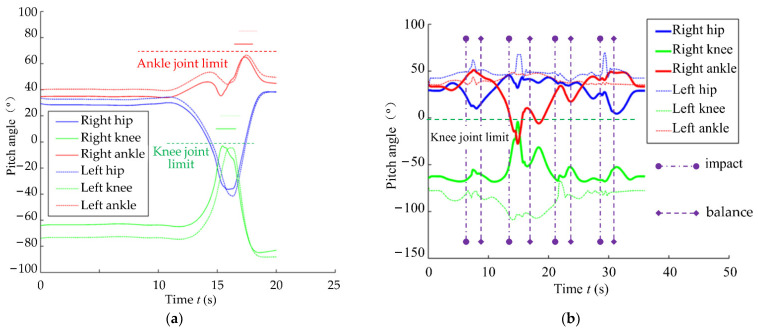
Pitch angles from two single-leg stance simulations. (**a**) Without impact; (**b**) with impact.

The joint angles in the same simulations are shown in Figure 12, wherein the joint limits are marked with horizontal lines. The moments of impact and restoration of equilibrium are also marked with vertical lines in Figure 12b. Figure 12a shows that the knee joints approach the joint limit between 15 s and 17 s, and the global self-stabilizer distributes the motion of the knee joints to the ankle joints.

**Figure 13:**
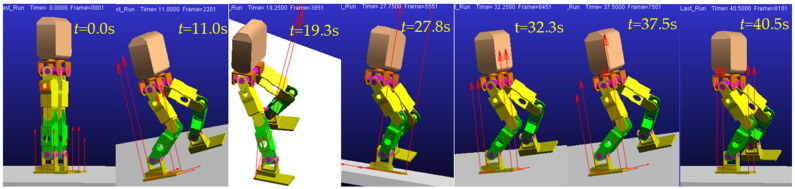
Screenshot of single-stance stability verification simulation with impact.

Screenshots of the single-stance stability verification simulation with impact using the virtual prototype in Adams are shown in Figure 13.

**Figure 14:**
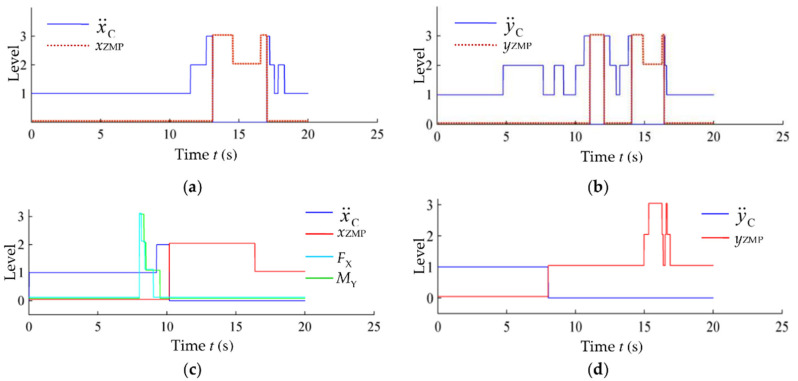
Action switching of the global self-stabilizer during single-leg stance. (**a**) Action switching on the *x*-axis without impact; (**b**) action switching on the *y*-axis without impact; (**c**) action switching on the *x*-axis with impact; (**d**) action switching on the *y*-axis with impact.

The action-switching process of the global self-stabilizer is given in Figure 14. The switching of ${\ddot{x}}_{C}$, ${\ddot{y}}_{C}$, *x*_ZMP_ and *y*_ZMP_ without impact are given in Figure 14a,b. When the sagittal impact is applied, the global self-stabilizer will use *F*_X_ and *M*_Y_ actions to replace ${\ddot{x}}_{F}$ and ${\ddot{\theta }}_{T1}$, respectively (for the lateral impact it will use *F*_Y_ and *M*_X_ to replace ${\ddot{y}}_{F}$ and ${\ddot{\theta }}_{T2}$, respectively). The switching process is shown in Figure 14c,d.

In the case of single-leg stance, the global self-stabilizer dynamically mixed TC and TB controllers in the case of no impact; in the presence of impact, the global self-stabilizer combined the four types of controllers, TC, TB, FE and TE. The controller parameters were also adjusted according to the system state. The switching rules were implicitly contained in value functions obtained from training process, and the results are equivalent to exploring different combinations of actions or parameters for calculating adjustments in different locations of the system space. Therefore, the global self-stabilizer obtained a stronger stability than the original controller used to generate the training data.

**Figure 15:**
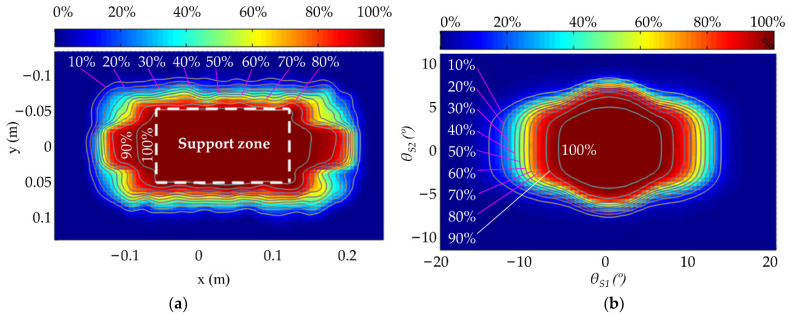
Success rate contour map of single-leg stance simulation with impact. (**a**) Success rate with respect to ZMP; (**b**) success rate with respect to flip angle.

The simulation data of single-leg stance with impact were sampled using a Gaussian function (standard deviation 5 mm). The probabilities of the distribution of the simulation success rate with respect to the ZMP position and the support surface flip angle are shown in Figure 15. From Figure 15a, it can be seen that the robot is basically guaranteed to be stable when the ZMP is within the support zone. The area circled by the contour with an 80% success rate is about 1.8 times the size of the support zone, indicating that the global self-stabilizer makes it possible for the robot to recover its balance even when the ZMP is out of the support zone. Figure 15b shows that the robot has 100% stability when the flip angle *θ_S1_* is less than 6° and *θ_S2_* is less than 5°; the success rate of recovering balance gradually decreases as the flip angle rises. Figure 15 also shows that the robot has stronger robustness to resist sagittal disturbances than lateral disturbances in single-leg stance.

**Figure 16:**
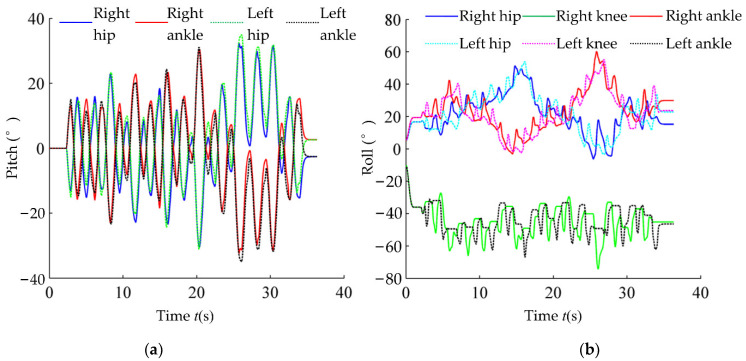
Joint angle in random stepping. (**a**) Roll joint angle; (**b**) pitch joint angle.

The joint angles in one stepping simulation are shown in Figure 16 which depicts that the robot has periodic trajectories of joint angles. In addition, there are also irregular fluctuations due to the changing ground tilt perturbation imposed by the moving platform.

**Figure 17:**
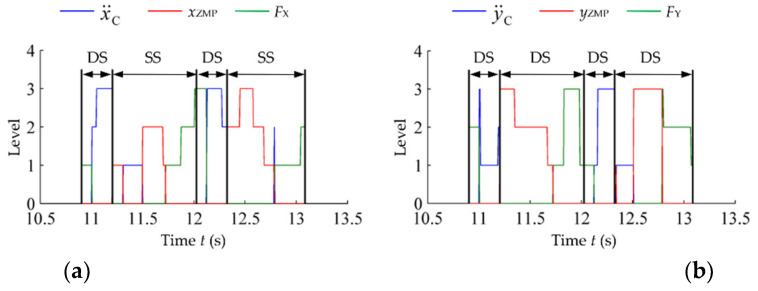
Action switching in random stepping. (**a**) Action switching in sagittal plane; (**b**) action switching in lateral plane.

The action switching in the sagittal and lateral planes are given in Figure 17. The action switching processes in two planes are similar. Where the CoM action is dominant during the double-legged stance period, the ZMP action is dominant during the single-leg stance period, and the inertial force action is used before and after the swing foot hits the ground.

## 6. Experiment Results

In this section, experiments are conducted firstly for stability training, followed by stability verification experiments using the trained global self-stabilizer to show the effects.

### 6.1. Stability Training Experiment

The global self-stabilizer obtained from the simulation was transplanted to the robot to reduce the wear of mechanical parts by frequent training experiments. The parameters that need to be transplanted include the parameter set ***Ψ*** of the basis function, the value function ***V*** and the connection weight matrix ***H****_i_* of the RBF network.

The stability training experiments of three motions were performed using the bipedal part of the GoRoBoT-II robot. The total number of experiments and the number of successes for each motion under different perturbation conditions are given in Table 9.

The transplanted global self-stabilizer was trained using the obtained experimental data, and the procedure and the parameters to be learned are similar to the simulation training in 5.1.

### 6.2. Stability Verification Experiment

For the three motions of double-leg stance, single-leg stance and stepping, twenty stability validation experiments were conducted, and the disturbances were generated according to the fourth, second, and first row parameters in Table 9, respectively. The corresponding success rates are 75%, 60% and 55%, respectively. Accordingly, the success rates of the model-based balance controller in the experiments were improved by 16.7%, 26.7% and 25.4%, respectively.

**Figure 18:**
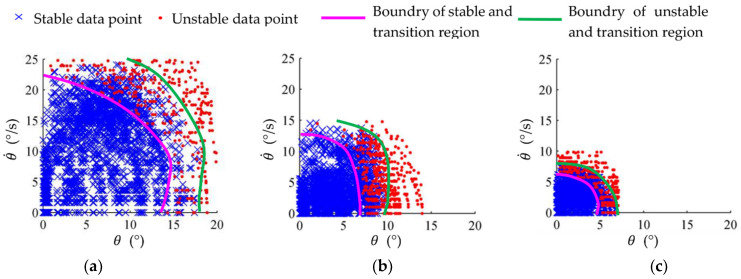
Data distributions in motion platform phase space. (**a**) Double-leg stance; (**b**) single-leg stance; (**c**) stepping.

The distributions of the experimental data in the platform phase space are shown in Figure 18, where the unstable points indicate that the robot met the unstable condition in Equation (19) within 3 s, while the stable points indicate that the robot did not fall over within 3 s. The phase space of the motion platform was divided into the stable region, the unstable region and the transition region. It can be seen that the stable region of all three motions is larger than the size of the unstable region and the transition region, indicating that the trained global self-stabilizer gained the ability to resist external perturbations.

**Figure 19:**
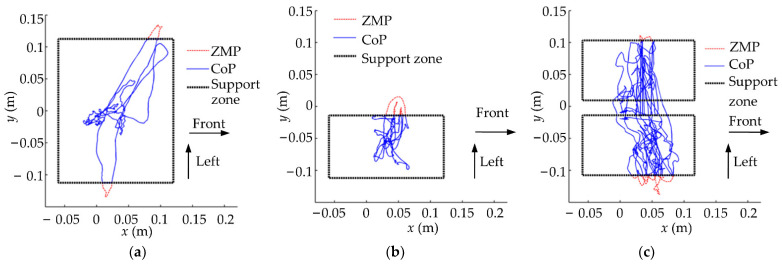
ZMP curves in three experiments. (**a**) Double-leg stance; (**b**) single-leg stance; (**c**) stepping.

**Figure 20:**
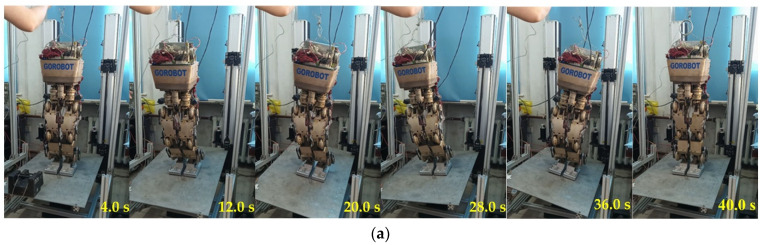

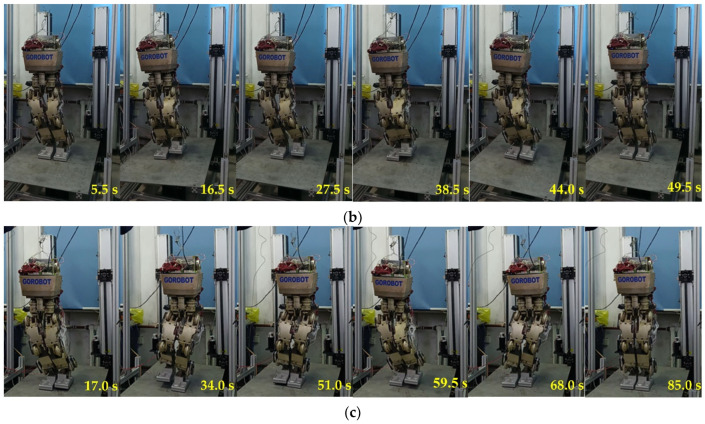
Screenshots of three experiments. (**a**) Double-leg stance; (**b**) single-leg stance; (**c**) stepping.

The ZMP curves that were obtained in three random experiments for each motion are given in Figure 19, which shows that the trained global stabilizer can restore balance even if the ZMP is out of the support zone. In addition, the corresponding experiment screenshots are shown in Figure 20.

In summary, the trained global self-stabilizer obtained the self-stabilization capability to cope with the random amplitude-limited perturbations under different motions. In addition, the stabilization capability was stronger than that of the model-based balance controllers after the training process, which indicates that the global self-stabilizer extracted and generated the control strategy that was most beneficial to maintain the robot’s balance based on the training data, and obtained a better state/action mapping.

## 7. Conclusions

A general model of a stability training system with a training platform is designed for legged robots with an arbitrary number of legs and an arbitrary configuration. The application of the proposed idea was given from three perspectives: system variable determination, action set construction and model-based controller designs for training data generation. A global self-stabilizer capable of learning from different sources of training data in a high-dimensional continuous system space was proposed to address the stability training problem of legged robots. The overall task of keeping the robot stable is broken down into three modules: action selection, adjustment calculation and joint motion mapping, in which the action selection and adjustment calculation modules use the Q-learning algorithm, and the joint motion mapping module uses a modified RBF network. 

Stability training simulations and experiments of the global self-stabilizer were conducted by taking the bipedal robot, GoRoBoT-II, as an example (it should also be noted that the application of the proposed training method was not limited by the size of robot). The training data that were generated from 18 controllers were used for training the global self-stabilizer.

Stability verification simulations and experiments were conducted for the trained global self-stabilizer, and the following conclusions can be obtained:

1. Simulation verification showed that the success rates of the trained global self-stabilizer, in three kinds of motion, under different disturbances, were higher than that of the model-based balance controller, with an improvement of at least 9.4%.
2. Experiment verification showed that the trained global self-stabilizer could keep the robot balanced under the random amplitude-limited tilt perturbation. The success rates of the stability verification experiments could reach 75%, 60% and 55%, respectively, which were higher than the success rates obtained using the model-based balance controller during the training data generation (58.3%, 33.3% and 29.6%, respectively).
3. The trained global self-stabilizer obtained different action combinations from the training data, and also continuously switched parameters according to the system state. This indicates that the designed global self-stabilizer was able to explore better state–action mapping from the training data and had the ability to learn and evolve continuously.

In summary, the proposed global self-stabilizer was able to accomplish the stability training task under compound perturbations and explore better action combinations from multiple different sources of training data. In the next step, we will put the trained global self-stabilizer into a real, unknown environment for further experiments.

## Figures and Tables

**Table 1:** Summary of learning-based balance control methods.

Scholar	Algorithm	State Space	Action Space	Disturbance
Scesa et al. [12]	CTRNN	6-d ^1^ continuous space	3-d continuous space	Sagittal/lateral push
Shieh et al. [13]	FNN	10-d continuous space	1-d continuous space	Tilt/rugged ground
Zhou et al. [14]	Fuzzy reinforcement learning	Two 2-d continuous space	1-d continuous space	None
Joao et al. [15]	SVM + FNN	2-d continuous space	1-d continuous space	None
Li et al. [16]	Fuzzy control + optimal control	Two 3-d continuous space	3-d continuous space	None
Hwang et al. [17]	Q-learning	82 discrete states	24 discrete actions	Seesaw
Hengst et al. [18]	Q-learning	4-d continuous space	9 discrete actions	None
Hwang et al. [19,20]	Q-learning + Reconstruction of Segmented Postures	8 discrete states	25 discrete actions	None
Liu et al. [21]	DDPG	4-d continuous space	2-d continuous space	Sagittal impact
Valle et al. [22]	Approximate Q-learning	66 discrete states	8 discrete actions	None
Li et al. [23]	PPO	20-d continuous space	10-d continuous space	Load of 15% total mass

^1^ short for 6-dimensional.

**Table 2:** System variables of a general model for stability training of legged robots.

Category of System Variables	Definition of Variable	Symbolic Representation
Joint motion	Angle, angular velocity and acceleration of joints	$\theta_{k}$$,\;{\dot{\theta }}_{k}$$,\;{\ddot{\theta }}_{k}$, *k* = 1, 2, … *N*_J_
Torso motion	Pose, velocity and acceleration of torso	$X_{T}\;=\;[x_{T},\;y_{T},\;z_{T},\;\theta_{T1},\;\theta_{T2},\;\theta_{T3}]{\;}^{T}{,}^{\;}{\dot{X}}_{T},\;{\ddot{X}}_{T}$
*j^th^* swing foot motion	Pose, velocity and acceleration of *j^th^* foot	$X_{Fj}\;=\;[x_{Fj},\;y_{Fj},\;z_{Fj},\;\theta_{Fj1},\;\theta_{Fj2},\;\theta_{Fj3}]{\;}^{T}{,}^{\;}{\dot{X}}_{Fj},\;{\ddot{X}}_{Fj}$
CoM motion	Pose, velocity and acceleration of CoM	$P_{C}\;=\;[x_{C},\;y_{C},\;z_{C}]{\;}^{T}{,}^{\;}{\dot{P}}_{C},\;{\ddot{P}}_{C}$
ZMP position	ZMP position in ΣO_B_	***P***_ZMP_ = [*x*_ZMP_, *y*_ZMP_, 0] ^T^
Inertial force and moment	Resultant force and moment at CoM	***F*** = [*F*_X_, *F*_Y_, *F*_Z_] ^T^ ***M*** = [*M*_X_, *M*_Y_, *M*_Z_] ^T^
Support zone flip motion	flip angle, angular velocity and angular acceleration of ΣO_S_ with respect to ΣO_P_	$\theta_{S1},\;{\dot{\theta }}_{S1}$ $,\;{\ddot{\theta }}_{S1},$ $\theta_{S2},\;{\dot{\theta }}_{S2}$ $,\;{\ddot{\theta }}_{S2}$
Moving platform motion	Pose, velocity and acceleration of ΣO_P_	$X_{P}{,}_{\;}{\dot{X}}_{P},\;{\ddot{X}}_{P}$

**Table 3:** Parameter table for action set.

Action	Parameter	Meaning
Single-joint action	*K*_11_, *K*_12_, *K*_13_	the compensation coefficients when the joint position, velocity and acceleration are close to the limit
	*ε*_11_, *ε*_12_, *ε*_13_	the width of the neighborhood where the joint position, velocity and acceleration start to avoid the limit
	*L* (·)	the compensation function for avoiding the joint limit
Torso action	$X_{T}^{d}$ $,\;{\dot{X}}_{T}^{d}$	the torso target pose and velocity vector
	*K*_21_, *K*_22_	the proportional and derivative coefficients for the torso adjustment
Swing foot action	$X_{Fj}^{d}$ $,\;{\dot{X}}_{Fj}^{d}$	the swing foot target pose and velocity vector
	*K*_31_, *K*_32_	the proportional and derivative coefficients for the swing foot adjustment
CoM action	***P***_S_, ***P***_C_	the position of the stance foot coordinate system origin O_S_ and the robot CoM
	*l*_C_	the distance from the robot CoM to the O_S_
	*K*_41_, *K*_42_	the proportional and derivative coefficients of the CoM adjustment
Inertial force/moment action	***F***_last_, ***M***_last_	the resultant inertial force and moment at the CoM in the last control cycle
	*m*_C_	the total mass of the robot
	***L***_C_	the angular momentum about the CoM
	*K*_51_, *K*_52_	the adjustment coefficients for the inertial force and moment
ZMP action	*x*_ZMP_, *y*_ZMP_	position of the ZMP point along the x and y axes within ΣO_S_
	***P***_CP_	the CP point position in the support zone
	***P***_0_	the position of the center point of stance foot
	*K*_6_	the coefficient for ZMP adjustment

**Table 4:** Main parameters of the biped robot GoRoBoT-II.

Parameter	Length (mm)	Parameter	Length (mm)	Parameter	Mass (kg)
Torso length *l*_0_	300	Hip width *l*_h_	125	Torso mass *m*_0_	12.5
Thigh length *l*_1_	220	Forefoot length *l*_f1_	120	Thigh mass *m*_1_	6
Calf length *l*_2_	189	Hindfoot length *l*_f2_	60	Calf mass *m*_2_	2.5
Ankle height *l*_3_	104	Foot width *l*_fw_	90	Foot mass *m*_3_	0.25

**Table 5:** Model-based balance controller for global self-stabilizer training data generation.

Balance Controller	Behavior	Symbol	Activated Action Variables
CoM motion	Torso translation	TC	$\Delta {\ddot{P}}_{C},\Delta {\ddot{\theta }}_{T1},\Delta {\ddot{\theta }}_{T2},\Delta {\ddot{\theta }}_{T3},\Delta {\ddot{X}}_{F}$
	*j*^*th*^ swing foot kick	FC	$\Delta {\ddot{X}}_{T},\Delta {\ddot{P}}_{C},\Delta {\ddot{\theta }}_{F1},\Delta {\ddot{\theta }}_{F2},\Delta {\ddot{\theta }}_{F3}$
Energy attenuation	Torso motion	TE	$\Delta F,\Delta M,\Delta {\ddot{X}}_{F}$
	*j*^*th*^ swing foot motion	FE	$\Delta {\ddot{X}}_{T},\Delta F,\Delta M$
CP balance control	Torso translation	TB	$\Delta x_{\mathrm{ZMP}},\Delta y_{\mathrm{ZMP}},\Delta {\ddot{z}}_{T},\Delta {\ddot{\theta }}_{T1},\Delta {\ddot{\theta }}_{T2},\Delta {\ddot{\theta }}_{T3},\Delta {\ddot{X}}_{F}$
	*j*^*th*^ swing foot kick	FB	$\Delta {\ddot{X}}_{T},\Delta x_{\mathrm{ZMP}},\Delta y_{\mathrm{ZMP}},\Delta {\ddot{z}}_{F},\Delta {\ddot{\theta }}_{F1},\Delta {\ddot{\theta }}_{F2},\Delta {\ddot{\theta }}_{F3}$

**Table 6:** Different levels of parameters for adjustment.

Action	Parameter	Value		
		Level 1	Level 2	Level 3
Single-joint action	(*K*_11_, *K*_12_, *K*_13_)	(5, 3, 1)	(4, 2, 1)	(3, 1, 1)
	(*ε*_1_, *ε*_2_, *ε*_3_)	(4°, 6°/s, 12°/s^2^)	(7°, 10°/s, 20°/s^2^)	(10°, 15°/s, 30°/s^2^)
Torso action	(*K*_21_, *K*_22_)	(14.1, 100)	(20, 100)	(28.2, 100)
Swing foot action	(*K*_31_, *K*_32_)	(14.1, 100)	(20, 100)	(28.2, 100)
CoM action	(*K*_41_, *K*_42_)	(14.1, 100)	(20, 100)	(28.2, 100)
Inertial force/moment action	(*K*_51_, *K*_52_)	(1, 0.5)	(1.5, 0.8)	(2, 1)
ZMP action	*K*_6_	0.8	1.2	1.6

**Table 7:** Results of key feature selection and autonomic abstraction calculation of state space.

	Variable	Selected State Variables	Number of Basis Function		
			Level 1	Level 2	Level 3
Action variable	$\Delta {\ddot{\theta }}_{Ri}$ (*i* = 1, 2, …,6)	$\theta_{Ri},\Delta {\dot{\theta }}_{Ri},\Delta {\ddot{\theta }}_{Ri}$	52 *	70 *	64 *
	$\Delta {\ddot{\theta }}_{Li}$ (*i* = 1, 2, …,6)	$\theta_{Li},\Delta {\dot{\theta }}_{Li},\Delta {\ddot{\theta }}_{Li}$	55 *	67 *	59 *
	$\Delta {\ddot{x}}_{T}$	*x*_T_, ${\dot{x}}_{T}$, *x*_ZMP_, *θ*_R5_, *θ*_R4_	1872	1935	1763
	$\Delta {\ddot{y}}_{T}$	*y*_T_, ${\dot{y}}_{T}$, *y*_ZMP_, *θ*_R6_	1119	1328	1266
	$\Delta {\ddot{z}}_{T}$	*z*_T_, ${\dot{z}}_{T}$, *θ*_R4_, ${\dot{\theta }}_{R4}$	1203	1298	1255
	$\Delta {\ddot{\theta }}_{T1}$	*θ*_P1_, ${\dot{\theta }}_{P1}$, *θ*_T1_, ${\dot{\theta }}_{T1}$	1353	1499	1296
	$\Delta {\ddot{\theta }}_{T2}$	*θ*_P2_, ${\dot{\theta }}_{P2}$, *θ*_T2_, ${\dot{\theta }}_{T2}$	1277	1394	1206
	$\Delta {\ddot{\theta }}_{T3}$	*θ*_T3_, ${\dot{\theta }}_{T3}$, *θ*_R1_	206	301	255
	$\Delta {\ddot{x}}_{F}$	*x*_F_, ${\dot{x}}_{F}$, *M*_Y_, *F*_X_, *θ*_S2_, ${\dot{\theta }}_{S2}$	2452	2571	2368
	$\Delta {\ddot{y}}_{F}$	*y*_F_, ${\dot{y}}_{F}$, *M*_X_, *F*_Y_, *θ*_S1_, ${\dot{\theta }}_{S1}$	2280	2246	2116
	$\Delta {\ddot{z}}_{F}$	*z*_F_, ${\dot{z}}_{F}$, *θ*_L4_, ${\dot{\theta }}_{L4}$	1368	1420	1297
	$\Delta {\ddot{\theta }}_{F1}$	*θ*_F1_, ${\dot{\theta }}_{F1}$, *θ*_L6_, ${\dot{\theta }}_{L6}$	1385	1538	1226
	$\Delta {\ddot{\theta }}_{F2}$	*θ*_F2_, ${\dot{\theta }}_{F2}$, *θ*_L5_, ${\dot{\theta }}_{L5}$	1235	1496	1126
	$\Delta {\ddot{\theta }}_{F3}$	*θ*_F3_, ${\dot{\theta }}_{F3}$, *θ*_L1_	341	391	335
	$\Delta {\ddot{x}}_{C}$	*x*_C_, ${\dot{x}}_{C}$, x_ZMP_, *θ*_S2_, ${\dot{\theta }}_{S2}$, *θ*_P2_, ${\dot{\theta }}_{P2}$, *M*_Y_, *F*_X_, *θ*_R5_, ${\dot{\theta }}_{R5}$	11,359	12,670	12,370
	$\Delta {\ddot{y}}_{C}$	*y*_C_, ${\dot{y}}_{C}$, *y*_ZMP_, *θ*_S1_, ${\dot{\theta }}_{S1}$, *θ*_P1_, ${\dot{\theta }}_{P1}$, *M*_X_, *F*_Y_, *θ*_R6_, ${\dot{\theta }}_{R6}$	12,697	13,019	11,268
	$\Delta {\ddot{z}}_{C}$	*z*_C_, ${\dot{z}}_{C}$, *θ*_R4_, ${\dot{\theta }}_{R4}$, *θ*_L4_, ${\dot{\theta }}_{L4}$	2332	2569	2571
	Δ*F*_X_	*F*_X_, *x*_ZMP_, *θ*_S2_, ${\dot{\theta }}_{S2}$, *θ*_P2_, ${\dot{\theta }}_{P2}$, *x*_C_, ${\dot{x}}_{C}$	5002	5233	5493
	Δ*F*_Y_	*F*_Y_, *y*_ZMP_, *θ*_S1_, ${\dot{\theta }}_{S1}$, *θ*_P1_, ${\dot{\theta }}_{P1}$, *y*_C_, ${\dot{y}}_{C}$	5540	5981	6127
	Δ*F*_Z_	*θ*_S2_, ${\dot{\theta }}_{S2}$, *θ*_S1_, ${\dot{\theta }}_{S1}$, *z*_C_, ${\dot{z}}_{C}$	3627	3826	3695
	Δ*M*_X_	*y*_ZMP_, *θ*_S1_, ${\dot{\theta }}_{S1}$, *θ*_P1_, ${\dot{\theta }}_{P1}$, *y*_C_, ${\dot{y}}_{C}$	3890	3452	3321
	Δ*M*_Y_	*x*_ZMP_, *θ*_S2_, ${\dot{\theta }}_{S2}$, *θ*_P2_, ${\dot{\theta }}_{P2}$, *x*_C_, ${\dot{x}}_{C}$	4023	3926	3751
	Δ*M*_Z_	*θ*_S2_, ${\dot{\theta }}_{S2}$, *θ*_S1_, ${\dot{\theta }}_{S1}$	1231	1396	1117
	Δ*x*_ZMP_	*x*_ZMP_, *θ*_P1_, ${\dot{\theta }}_{P1}$, *θ*_P2_, ${\dot{\theta }}_{P2}$, *θ*_S1_, *θ*_S2_, *x*_C_, ${\dot{x}}_{C}$, *y*_C_, ${\dot{y}}_{C}$	8695	9007	9861
	Δ*y*_ZMP_	*y*_ZMP_, *θ*_P1_, ${\dot{\theta }}_{P1}$, *θ*_P2_, ${\dot{\theta }}_{P2}$, *θ*_S1_, *θ*_S2_, *x*_C_, ${\dot{x}}_{C}$, *y*_C_, ${\dot{y}}_{C}$	8824	8937	9331
Joint mapping	Δ*F*_X_	*x*_C_, *z*_C_, *θ*_R1_, *θ*_R2_, *θ*_R3_, *θ*_R4_, *θ*_R5_, *θ*_R6_	6892		
	Δ*F*_Y_	*y*_C_, *z*_C_, *θ*_R1_, *θ*_R2_, *θ*_R3_, *θ*_R4_, *θ*_R5_, *θ*_R6_	7101		
	Δ*F*_Z_	*x*_C_, *y*_C_, *z*_C_, *θ*_R1_, *θ*_R2_, *θ*_R3_, *θ*_R4_, *θ*_R5_, *θ*_R6_	7840		
	Δ*x*_ZMP_	*x*_C_, *z*_C_, *θ*_R1_, *θ*_R2_, *θ*_R3_, *θ*_R4_, *θ*_R5_, *θ*_R6_, *θ*_L2_, *θ*_L3_, *F*_X_, *F*_Z_, *M*_Y_	24,427		
	Δ*y*_ZMP_	*y*_C_, *z*_C_, *θ*_R1_, *θ*_R2_, *θ*_R3_, *θ*_R4_, *θ*_R5_, *θ*_R6_, *θ*_L2_, *θ*_L3_, *F*_Y_, *F*_Z_, *M*_X_	22,246		

* Denotes the average number of basis functions.

**Table 8:** Comparison of the simulation success rates of the trained global self-stabilizer and model-based balance controllers.

Motion	Success Rate without Impact		Success Rate with Impact	
	Global Self-Stabilizer	Model-Based Controllers	Global Self-Stabilizer	Model-Based Controllers
Double-leg stance	97.4%	88% (max)	85.7%	47% (max)
Single-leg stance	94.2%	75% (max)	80.2%	47% (max)
Stepping	76.6%	44% (max)	-	-

**Table 9:** Parameters and results of stability training experiments.

Platform Moving Parameter (Angle, Angular Velocity and Acceleration)	Success/Overall		
	Double-Leg Stance	Single-Leg Stance	Stepping
±7° ±10°/s, ±20°/s^2^	11/12	17/24	16/54
±14° ±15°/s, ±30°/s^2^	17/24	10/30	-
±20° ±20°/s, ±40°/s^2^	7/12	-	-
±20° ±25°/s, ±60°/s^2^	2/6	-	-

## References

[1] Borovac B., Vukobratovic M., Surla D. (1989). An Approach to Biped Control Synthesis. Robotica.

[2] Yokoi K., Kanehiro F., Kaneko K., Kajita S., Fujiwara K., Hirukawa H. (2004). Experimental study of humanoid robot HRP-1S. Int. J. Robot Res..

[3] Hirukawa H., Kanehiro F., Kajita S., Fujiwara K., Yokoi K., Kaneko K., Harada K. (2003). Experimental evaluation of the dynamic simulation of biped walking of humanoid robots. Proceedings of the 20th IEEE International Conference on Robotics and Automation (ICRA).

[4] Okada K., Ogura T., Haneda A., Inaba M. (2005). Autonomous 3D walking system for a humanoid robot based on visual step recognition and 3D foot step planner. Proceedings of the IEEE International Conference on Robotics and Automation (ICRA).

[5] Kim J.W., Tran T.T., Dang C.V., Kang B. (2016). Motion and Walking Stabilization of Humanoids Using Sensory Reflex Control. Int. J. Adv. Robot Syst..

[6] Kaewlek N., Maneewarn T. (2005). Inclined Plane Walking Compensation for a Humanoid Robot. Proceedings of the International Conference on Control, Automation and Systems (ICCAS 2010).

[7] Yang S.P., Chen H., Fu Z., Zhang W. (2021). Force-feedback based Whole-body Stabilizer for Position-Controlled Humanoid Robots. Proceedings of the IEEE/RSJ International Conference on Intelligent Robots and Systems (IROS), Electr Network.

[8] Seo K., Kim J., Roh K. (2012). Towards Natural Bipedal Walking: Virtual Gravity Compensation and Capture Point Control. Proceedings of the 2012 IEEE/RSJ International Conference on Intelligent Robots and Systems.

[9] Elhasairi A., Pechev A. (2015). Humanoid robot balance control using the spherical inverted pendulum mode. Front. Robot AI.

[10] Alcaraz-Jimenez J.J., Herrero-Perez D., Martinez-Barbera H. (2013). Robust feedback control of ZMP-based gait for the humanoid robot Nao. Int. J. Robot Res..

[11] Gao L.Y., Wu W.G., Ieee Kinetic Energy Attenuation Method for Posture Balance Control of Humanoid Biped Robot under Impact Disturbance. Proceedings of the 44th Annual Conference of the IEEE Industrial-Electronics-Society (IECON).

[12] Henaff P., Scesa V., Ben Ouezdou F., Bruneau O. (2011). Real time implementation of CTRNN and BPTT algorithm to learn on-line biped robot balance: Experiments on the standing posture. Control Eng. Pract..

[13] Shieh M.Y., Chang K.H., Chuang C.Y., Lia Y.S., Ieee Development and implementation of an artificial neural network based controller for gait balance of a biped robot. Proceedings of the 33rd Annual Conference of the IEEE-Industrial-Electronics-Society.

[14] Zhou C.J., Meng Q.C. (2003). Dynamic balance of a biped robot using fuzzy reinforcement learning agents. Fuzzy Sets Syst..

[15] Ferreira J.P., Crisostomo M.M., Coimbra A.P. (2009). SVR Versus Neural-Fuzzy Network Controllers for the Sagittal Balance of a Biped Robot. IEEE Trans. Neural Netw..

[16] Li Z.J., Ge Q.B., Ye W.J., Yuan P.J. (2016). Dynamic Balance Optimization and Control of Quadruped Robot Systems With Flexible Joints. IEEE Trans. Syst. Man Cybern. Syst..

[17] Hwang K.S., Li J.S., Jiang W.C., Wang W.H. (2013). Gait Balance of Biped Robot based on Reinforcement Learning. Proceedings of the SICE Annual Conference.

[18] Hengst B., Lange M., White B. Learning ankle-tilt and foot-placement control for flat-footed bipedal balancing and walking. Proceedings of the 2011 11th IEEE-RAS International Conference on Humanoid Robots.

[19] Lin J.L., Hwang K.S. (2017). Balancing and Reconstruction of Segmented Postures for Humanoid Robots in Imitation of Motion. IEEE Access.

[20] Hwang K.S., Jiang W.C., Chen Y.J., Shi H.B. (2017). Motion Segmentation and Balancing for a Biped Robot’s Imitation Learning. IEEE Trans. Ind. Inform..

[21] Liu C.J., Lonsberry A.G., Nandor M.J., Audu M.L., Lonsberry A.J., Quinn R.D. (2019). Implementation of Deep Deterministic Policy Gradients for Controlling Dynamic Bipedal Walking. Biomimetics.

[22] Valle C.M.C.O., Tanscheit R., Mendoza L.A.F. Computed-Torque Control of a Simulated Bipedal Robot with Locomotion by Reinforcement Learning. Proceedings of the 2016 IEEE Latin American Conference on Computational Intelligence (La-Cci).

[23] Li Z.Y., Cheng X.X., Peng X.B., Abbeel P., Levine S., Berseth G., Sreenath K., Ieee Reinforcement Learning for Robust Parameterized Locomotion Control of Bipedal Robots. Proceedings of the IEEE International Conference on Robotics and Automation (ICRA).

[24] Wu W.G., Du W.Q. (2014). Research of 6-DOF Serial-Parallel Mechanism Platform for Stability Training of Legged-Walking Robot. J. Harbin Inst. Technol. (New Ser.).

[25] Wu W.G., Gao L.Y. (2017). Posture self-stabilizer of a biped robot based on training platform and reinforcement learning. Robot Auton. Syst..

[26] Jelsma D., Ferguson G.D., Smits-Engelsman B.C.M., Geuze R.H. (2015). Short-term motor learning of dynamic balance control in children with probable Developmental Coordination Disorder. Res. Dev. Disabil..

[27] Maciaszek J., Borawska S., Wojcikiewicz J. (2014). Influence of Posturographic Platform Biofeedback Training on the Dynamic Balance of Adult Stroke Patients. J. Stroke Cerebrovasc. Dis..

[28] DiFeo G., Curlik D.M., Shors T.J. (2015). The motirod: A novel physical skill task that enhances motivation to learn and thereby increases neurogenesis especially in the female hippocampus. Brain Res..

[29] Wu W.G., Gao L.Y. (2021). Modular combined motion platform used for stability training and amplitude limiting random motion planning and control method. CN Patent.

[30] Gao L.Y., Wu W.G. (2020). Relevance assignation feature selection method based on mutual information for machine learning. Knowl.-Based Syst..

[31] Hou Y.Y. (2014). Research on Flexible Drive Unit and Its Application in Humanoid Biped Robot. Ph.D. Dissertation.

